# Investigating associations between emotional and behavioral problems, self-esteem, and parental attachment among adolescents: A cross-sectional study in Indonesia

**DOI:** 10.1016/j.heliyon.2023.e21459

**Published:** 2023-10-30

**Authors:** Rika Sarfika, I Made Moh Yanuar Saifudin, Ira Mulya Sari, Dewi Murni, Hema Malini, Khatijah Lim Abdullah

**Affiliations:** a Department of Mental Health and Community, Faculty of Nursing, Universitas Andalas, Padang, Indonesia; b Doctoral Student, Doctor of Medicine and Health, Faculty of Medicine, Public Health and Nursing, Universitas Gadjah Mada, Yogyakarta, Indonesia; c Department of Maternity and Child, Faculty of Nursing, Universitas Andalas, Padang, Indonesia; d Department of Basic Nursing, Faculty of Nursing, Universitas Andalas, Padang, Indonesia; e Department of Medical Surgical, Faculty of Nursing, Universitas Andalas, Padang, Indonesia; f Department of Nursing, School of Medical and Life Sciences, Sunway University, Selangor, Malaysia

**Keywords:** Adolescents, Emotional and behavioral problems, Parental attachment, Self-esteem

## Abstract

**Background:**

Emotional and Behavioral Problems (EBPs) are prevalent among adolescents, and adolescents’ capacity for adaptation can be influenced by their interactions with their parents, environment, and self-esteem. This link has not been systematically examined among adolescents in West Sumatra, Indonesia. This study aimed to assess the association of parental attachment and self-esteem with EBPs in adolescents.

**Methods:**

A cross-sectional study was conducted from July to November 2022 in Padang West Sumatra, Indonesia. In total, 854 students from public senior high school 4 Padang were involved in this study and completed questionnaires on demographics, EBPs, parental attachment, and self-esteem. There was a total of five subscales for EBPs, which included emotional problems, conduct problems, hyperactivity, peer problems and prosocial. Additionally, there were three subscales for parental attachment, which included the mother's attachment, father's attachment, and peer attachment. Spearman's correlation, independent-sample t-tests, ANOVA and multiple linear regression analysis were employed to examine factors associated with EBPs.

**Results:**

This study showed that father's attachment (r = −0.191, p < 0.001), mother's attachment (r = −0.241, p < 0.001), and self-esteem (r = −0.437, p < 0.001) were negatively correlated with EBPs. The linear regression analysis showed EBPs was associated with father's education, father's communication, father's alienation, mother's alienation, and self-esteem. All predictors of overall EBPs among adolescents were able to explain 31 % of the variance in EBPs.

**Conclusion:**

High self-esteem and a strong parental attachment have positive outcomes in terms of mental health in adolescents. Thus, increasing adolescent self-esteem and establishing a warm parent attachment can be the main target in providing interventions for Indonesian adolescents with EBPs.

## Highlights

• Father's attachment, mother's attachment, and self-esteem were all negatively correlated with EBPs, emphasizing the importance of these factors in adolescent mental health.
• Factors such as father's education, father's communication, father's alienation, mother's alienation, and self-esteem were identified as predictors of EBPs.
• The interventions aimed at enhancing self-esteem and promoting warm parental attachment could be key strategies for addressing EBPs in Indonesian adolescents.

## 1 Background

Adolescence frequently witnesses the occurrence of mental health challenges, encompassing various emotional and behavioral disorders such as disruptive behavior, depression, anxiety, and pervasive developmental disorders like autism. In Indonesia, the prevalence of Emotional and Behavioral Problems (EBPs) among adolescents aged 12 to 18 in Indonesia stands at 28.4 %. These problems encompass emotional issues (29.2 %), hyperactivity (8.4 %), conduct disorders (32.9 %), and peer-related problems (13.1 %) [1]. These disorders are often categorized as either internalizing or externalizing problems [2]. Previous research studies have reported that the prevalence of these Emotional and Behavioral Problems (EBPs) globally among children and adolescents falls within the range of 9.3 %–28.4 % [[3], [4], [5], [6], [7]]. It was found that higher levels of overall stress, particularly stress related to school and future prospects, were linked to a greater occurrence of emotional and behavioral problems [8].

Persistent Emotional and Behavioral Problems (EBPs) pose an elevated risk of causing both societal and individual challenges [3]. In adolescents, these EBPs have significant adverse impacts on society, resulting in direct behavioral consequences and associated costs. Moreover, they adversely affect the affected adolescents themselves, leading to difficulties in academic performance, occupational prospects, and psychosocial functioning. Families of these adolescents are also impacted [2,9].

EBPs often come with a high level of co-occurring conditions, hindered development of social skills, an increased likelihood of involvement in criminal activities, and a heightened risk of suicide. To mitigate these issues, it is imperative to gain a comprehensive understanding of the factors that contribute to the development of these problems [10,11].

The adaptability of adolescents is closely linked to their interactions with their parents, the actions of their parents, and the environment they are in Ref. [12]. The emotional development and mental well-being of adolescents are directly shaped by their parents' influence [13,14]. Furthermore, the attitudes of parents and their manner of interacting with adolescents play a significant role in determining how effectively they function. This, in turn, indirectly impacts their ability to adapt [15,16].

Various factors can influence parents' reports on Emotional and Behavioral Problems (EBPs), including age, gender, parental education, socioeconomic status, the level of support from parents, and the quality of the parent-child relationship [17,18]. Research has also demonstrated differences in reports between parents and children at different ages [19], between reports from fathers and mothers [20], and between reports from teachers and parents [21]. Many of the reasons for these disparities in reporting seem to be linked to environmental and external factors [22,23].

Additionally, self-esteem and resilience are crucial components of good mental health [[24], [25], [26]]. Moreover, happiness is recognized as a key measure of a person's hedonistic perspective or emotional well-being, encompassing positive affectivity and life satisfaction [[27], [28], [29]].

Building upon the aforementioned findings, the primary aim of this study was to examine the connection between parent-adolescent relationships and self-esteem concerning emotional and behavioral problems in adolescents. The principal contribution of this research lies in its exploration of parent-adolescent relationships and self-esteem as potential protective factors that could help alleviate the effects of mental health difficulties among adolescents. Importantly, these factors are dynamic and subject to change, especially during the adolescent years, which underscores their potential relevance in the design of prevention and intervention programs aimed at addressing mental health issues among adolescents.

## 2 Methods

### 2.1 Study design

The correlative descriptive study with cross-sectional design was used.

### 2.2 Participants

The data for this study were gathered from members of the Minangkabau ethnic group attending Public Senior High School (PSHS) 4 in Padang, located in West Sumatra, Indonesia. The participants were recruited in collaboration with the school. To be eligible for the initial study, students needed to meet the following criteria: be between the ages of 12–20 years old, be in grades 10th through 12th, and express a willingness to participate. Those who had significant difficulties in completing the questionnaire due to physical or psychological issues during the study were excluded. Consecutive sampling method was use to collect the data. The sample size was determined using G*Power 3.1, which suggested that 822 participants are needed for multiple linear regression with twelve independent variables, assuming an effect size of 0.05, an alpha level of 0.01, and 99 % power, however, we collected responses from 854 individuals who completed the survey comprehensively. Ultimately, no missing data were found in the final analysis.

### 2.3 Measures

In this research, four instruments were employed. The initial instrument comprised demographic variables, including gender, age, grade level, residential status, parental education, parental occupation, and family income. The other instruments used in the study were the Strengths and Difficulties Questionnaire (SDQ), the Inventory of Parent and Peer Attachment (IPPA), and the Rosenberg Self-Esteem Scale (RSES). Furthermore, the researchers obtained permission from the developer of the Indonesian version of these questionnaires to utilize them in this study. A description of each instrument is provided below.

1) Emotional and Behavioral Problems (EBPs)

To measure emotional and behavioral problems, the Strengths and Difficulties Questionnaire (SDQ) developed by Ref. [30] was used. This questionnaire has been adapted into Indonesian by Refs. [31,32] and has subsequently been established by the Ministry of Health, Republic of Indonesia to screen for EBPs among Indonesian adolescents. This instrument comprises 25 items designed to assess five different subscales related to EBPs. These subscales include emotional problems (e.g., “Often complains of headaches”), conduct problems (e.g., “Often has temper tantrums or hot tempers”), hyperactivity (e.g., “Constantly fidgeting or squirming”), peer problems (e.g., “Rather solitary, tends to play alone”), and prosocial behavior (e.g., “Considerate of other people's feelings”). Each response is rated on a three-point scale (0 = not true, 1 = somewhat true, and 2 = extremely true). To calculate the overall score for each subscale, one can add up the scores from all relevant items. Each subscale has a possible maximum score of 10 and a minimum score of 0. The instrument demonstrated strong overall item reliability with a high Cronbach's α score of 0.863. Furthermore, each individual scale within the instrument also exhibited satisfactory Cronbach's α scores, including emotional problems (0.804), conduct problems (0.486), hyperactivity (0.753), peer problems (0.513), and prosocial (0.852).

2) Parental Attachment

The Inventory of Parent and Peer Attachment (IPPA) was used to assess parental attachment. The questionnaire was developed by Ref. [33]. Indonesian version of this instrument was adopted by previous study [34,35]. The measurement is divided into three subscales, namely, 25 items for the mother's attachment subscale, 25 for the father's attachment subscale, and another 25 for the peer attachment subscale. In this study, we were only interested in the parental attachment subscales (father's and mother's attachment). The respondents were required to indicate the extent to which each item was true for them on a five-point scale (1 = never true; 5 = always true). The items on scales for mother and father are clustered into three factors (trust, communication, and alienation) by principal components analysis. Trust refers to mutual trust and respect for each other's needs and desires (e.g., My father accept me as I am). Communication refers to the perceived quality of involvement, responsiveness, and verbal communication related to an adolescent's emotional states (e.g., My father can tell when I'm upset about something). Alienation involves feeling socially isolated, angry, and detached from a parent, but at the same time feeling the need to be closer to them (e.g., I feel alone or apart when I am with my father). The Cronbach's alpha score for father's attachment was 0.890 and for mother's attachments was 0.870.

3) Self-esteem

Self-esteem was measured with a short version of the Rosenberg Self-Esteem Scale (RSES) [36]. The Indonesian version of RSES was adapted by previous study [37]. The RSES is a self-rating scale comprising five negative and five positive items. The respondents answered on a four-point scale ranging from “I totally agree” (1) to “I totally disagree” (4). High scores correspond to high self-esteem [38]. The Cronbach's alpha score of this instrument was 0.899.

### 2.4 Ethical considerations

Ethical approval for the study was obtained from the Research Ethics Committee of the Faculty of Medicine at Universitas Andalas, Padang, Indonesia (Approval number: 847/UN.16.2/KEP-FK/2022). Furthermore, the school approved to conduct the study, and the parents gave the student's consent through the guidance counselor's assistance. All the participants were fully informed about the aim of the study and how the data was used and stored, besides confirming that the researcher complied with all relevant ethical regulations to maintain the confidentiality of the participants' information. All participants provided informed consent before filling out the instruments. Additionally, anonymization was performed in data collection.

### 2.5 Statistical analyses

All statistical analyses were conducted using IBM SPSS version 22 (IBM Corp., Armonk, N·Y., USA). Demographic variables such as gender, age, grade level, residential status, parental education, parental occupation, and family income were expressed as percentages, means, and standard deviations. To assess the normality of continuous variables, the Kolmogorov-Smirnov test was employed. Group comparisons were made using independent-sample t-tests and ANOVA. The relationships between research variables were explored through Spearman's correlations. Statistical significance was considered when *p*-values were <0.05 (two-sided). Furthermore, multiple linear regression analysis was employed to identify the factors that influenced Emotional Behavioral Problems (EBPs).

## 3 Results

### 3.1 Characteristics of the participants and study variables

Table 1 presents the socio-demographics of the respondents. This study included 854 adolescents, consisting of 380 males (44.5 %) and 474 females (55.5 %). Most respondents were middle adolescents (61.4 %), and about 39.0 % were in grade 10. Almost all participants lived with their parents (94.3 %). Most parents completed senior high school education (father = 519 [60.8 %], mother = 486 [56.9 %]). The majority of mothers were unemployed (71.3 %). Most of the participants lived with a household income under the regional minimum wage (63.1 %).

**Table 1:** Characteristic of participants (n = 854).

Variables	*f*	%
Gender		
Male	380	44.5
Female	474	55.5
**Age (year)**		
Early adolescent (12–14)	13	1.5
Middle adolescent (15–17)	524	61.4
Late adolescent (18–20)	317	37.1
**Class level**		
Grades 10	333	39.0
Grades 11	281	32.9
Grades 12	240	28.1
**Residence status**		
With parents	805	94.3
With stepparents	5	0.6
With other family	40	4.7
Living alone	4	0.5
**Father's Education**		
No formal education	13	1.5
Primary School	57	6.7
Junior high school	116	13.6
Senior high school	519	60.8
College	149	17.4
**Mother's Education**		
No formal education	15	1.8
Primary School	65	7.6
Junior high school	101	11.8
Senior high school	486	56.9
College	187	21.9
**Father's Occupational**		
Civil servant	97	11.4
State employee	14	1.6
Private employees	85	10.0
Self-employed	245	28.7
Outsourcing	16	1.9
Retiring	31	3.6
Unemployment	38	4.4
Others	328	38.4
**Mother's Occupational**		
Civil servant	81	9.5
State employee	2	0.2
Private employees	23	2.7
Self-employed	100	11.7
Outsourcing	10	1.2
Retiring	9	1.1
Unemployment	609	71.3
Others	20	2.3
**Family Income**		
< Regional minimum wage	539	63.1
> Regional minimum wage	315	36.9

### 3.2 EBPs and their differences among socio-demographic groups

Table 2 provides an overview of the mean scores of the total and individual subscales of EBPs. The overall mean score of the total EBPs among participants was 24.15 (SD = 5.82), with scores ranging from 0 (no or mild EBPs) to 50 (severe EBPs). For the emotional problems subscale and the conduct problems subscale the mean scores were 5.28 (SD = 2.66), with scores ranging from 0 (no or mild emotional symptoms) to 10 (severe emotional symptoms); and 3.33 (SD = 1.59), with scores ranging from 0 (no or mild conduct problems) to 10 (severe conduct problems) respectively. For the hyperactivity-inattention subscale, the mean score was 4.20 (SD = 1.79), with scores ranging from 0 (no or mild hyperactivity-inattention problems) to 10 (severe hyperactivity-inattention problems), while for the peer problems subscale, the mean score was 3.74 (SD = 1.82), with scores ranging from 0 (no or mild peer relationship problems) to 10 (severe peer relationship problems). For the prosocial behavior subscale, the mean score was 7.62 (SD = 1.85), with scores ranging from 0 (no or low prosocial behavior) to 10 (high prosocial behavior).

**Table 2:** Differences EBPs according to characteristic.

Variables	EBPs Total			EBPs Emotional Problems			EBPs Conduct Problems			EBPs Hyperactivity			EBPs Peer Problems			EBPs Prosocial		
	Mean ± SD	t/F	p value	Mean ± SD	t/F	p value	Mean ± SD	t/F	p value	Mean ± SD	t/F	p value	Mean ± SD	t/F	p value	Mean ± SD	t/F	p value
Gender^a^																		
Male	23.01 ± 5.71	−5.19	<.001***	4.16 ± 2.43	−11.99	<.001***	3.56 ± 1.65	4.20	<.001***	4.05 ± 1.70	−2.20	.028*	3.77 ± 1.78	0.45	.652	7.47 ± 1.87	−2.13	.033*
Female	25.06 ± 5.75			6.18 ± 2.49			3.10 ± 1.52			4.32 ± 1.86			3.72 ± 1.85			7.74 ± 1.83		
**Age**^**b**^																		
Early adolescent (12–14)	25.92 ± 4.75	0.68	.509	6.00 ± 3.24	1.15	.316	3.08 ± 1.55	0.13	.877	4.38 ± 1.94	0.36	.699	5.23 ± 1.88	4.55	.011*	7.23 ± 1.30	1.93	.145
Middle adolescent (15–17)	24.17 ± 5.88			5.24 ± 2.62			3.31 ± 1.61			4.21 ± 1.79			3.71 ± 1.81			7.59 ± 1.85		
Late adolescent (18–20)	24.03 ± 5.77			5.60 ± 2.96			3.31 ± 1.49			4.05 ± 1.78			3.81 ± 1.88			7.99 ± 1.91		
**Class level**^**b**^																		
Grades 10	23.93 ± 5.69	0.53	.588	5.09 ± 2.64	1.44	.236	3.28 ± 1.64	1.36	.256	4.14 ± 1.68	0.86	.424	3.74 ± 1.76	2.14	.118	7.67 ± 1.81	0.96	.382
Grades 11	24.42 ± 6.17			5.44 ± 2.73			3.42 ± 1.62			4.16 ± 1.88			3.89 ± 1.86			7.49 ± 1.91		
Grades 12	24.13 ± 5.59			5.35 ± 2.61			3.19 ± 1.50			4.33 ± 1.83			3.56 ± 1.84			7.70 ± 1.85		
**Residence status**^**b**^																		
With parents	24.06 ± 5.75	4.39	.004**	5.24 ± 2.66	2.23	.083	3.30 ± 1.56	2.48	.060	4.21 ± 1.80	1.87	.132	3.70 ± 1.78	5.27	.001**	7.62 ± 1.84	0.97	.405
With stepparents	24.90 ± 6.08			6.00 ± 2.58			3.28 ± 2.09			3.83 ± 1.55			4.20 ± 2.24			7.60 ± 2.02		
With other family	33.20 ± 6.80			7.40 ± 2.61			5.00 ± 1.41			5.60 ± 1.14			6.40 ± 1.82			8.80 ± 2.17		
Living alone	23.50 ± 8.58			4.50 ± 3.32			2.25 ± 1.26			5.00 ± 2.16			5.00 ± 2.16			6.75 ± 0.96		
**Father's Education**^**b**^																		
No formal education	25.62 ± 6.11	1.04	.384	5.85 ± 2.27	0.91	.455	3.31 ± 1.65	2.33	.055	4.31 ± 1.65	0.32	.863	4.54 ± 1.33	1.14	.334	7.62 ± 1.94	0.59	.669
Primary School	24.44 ± 5.62			4.93 ± 2.59			3.72 ± 1.76			4.42 ± 1.78			3.95 ± 1.47			7.42 ± 1.86		
Junior high school	24.91 ± 6.48			5.55 ± 2.86			3.55 ± 1.58			4.23 ± 1.66			3.85 ± 1.07			7.72 ± 1.60		
Senior high school	24.04 ± 5.69			5.30 ± 2.66			3.25 ± 1.58			4.16 ± 1.79			3.67 ± 1.80			7.66 ± 1.90		
College	23.66 ± 5.78			5.08 ± 2.56			3.11 ± 1.56			4.23 ± 1.90			3.77 ± 1.83			7.46 ± 1.86		
**Mother's Education**^**b**^																		
No formal education	23.87 ± 6.52	1.01	.400	5.27 ± 2.66	1.16	.325	3.20 ± 1.90	2.15	.073	4.60 ± 1.88	0.36	.836	3.67 ± 1.50	0.627	.644	7.13 ± 1.88	0.95	.436
Primary School	25.46 ± 5.88			5.60 ± 2.55			3.62 ± 1.57			4.31 ± 2.04			4.06 ± 1.83			7.88 ± 1.76		
Junior high school	23.98 ± 5.66			4.95 ± 2.765			3.62 ± 1.51			4.21 ± 1.77			3.79 ± 1.76			7.41 ± 1.80		
Senior high school	24.14 ± 5.99			5.39 ± 2.69			3.25 ± 161			4.15 ± 1.74			3.72 ± 1.86			7.62 ± 1.85		
College	23.81 ± 5.35			5.06 ± .254			3.17 ± 1.56			4.25 ± 1.86			3.66 ± 1.77			7.67 ± 1.90		
**Father's Occupation**^**b**^																		
Civil servant	23.71 ± 5.94	4.03	<.001***	5.06 ± 2.74	3.53	.001**	3.21 ± 1.55	2.08	.043*	4.19 ± 2.09	0.63	.734	3.64 ± 1.88	2.38	.020*	7.62 ± 1.97	2.37	.021*
State employee	19.57 ± 533			2.79 ± 2.08			2.71 ± 1.38			3.93 ± 1.77			2.86 ± 1.87			7.29 ± 1.98		
Private employees	25.93 ± 5.14			5.89 ± 2.60			3.51 ± 1.56			4.53 ± 1.62			4.26 ± 1.75			7.74 ± 1.79		
Self-employed	24.36 ± 5.80			5.33 ± 2.63			3.40 ± 1.58			4.14 ± 1.78			3.88 ± 1.88			7.60 ± 1.83		
Outsourcing	21.50 ± 5.38			4.25 ± 2.77			2.19 ± 1.27			4.38 ± 2.06			3.00 ± 2.06			7.69 ± 1.81		
Retiring	21.65 ± 5.73			4.48 ± 2.10			3.26 ± 1.77			4.06 ± 1.75			3.48 ± 1.78			6.35 ± 1.92		
Unemployment	25.08 ± 5.50			5.79 ± 2.47			3.63 ± 1.85			4.39 ± 1.42			3.50 ± 1.84			7.76 ± 1.76		
Others	24.11 ± 5.86			5.32 ± 2.69			3.25 ± 1.58			4.16 ± 1.78			3.66 ± 1.73			7.72 ± 1.82		
**Mother's Occupation**^**b**^																		
Civil servant	23.68 ± 5.55	0.85	.545	5.06 ± 2.67	2.17	.035*	3.20 ± 1.45	2.19	.033*	4.05 ± 1.88	0.41	.896	3.73 ± 1.63	0.69	.676	7.64 ± 1.93	1.99	.053
State employee	19.00 ± 4.24			2.00 ± 0.00			5.00 ± 1.41			3.50 ± 2.12			2.50 ± 2.12			6.00 ± 1.41		
Private employees	25.83 ± 5.10			6.78 ± 2.17			3.83 ± 1.85			4.52 ± 1.73			3.65 ± 1.64			7.04 ± 2.01		
Self-employed	24.18 ± 5.85			5.16 ± 2.53			3.48 ± 1.54			4.24 ± 1.74			3.84 ± 1.87			7.46 ± 1.95		
Outsourcing	23.50 ± 4.72			4.40 ± 2.59			2.50 ± 1.90			4.40 ± 1.26			3.80 ± 1.55			8.40 ± 1.51		
Retiring	24.11 ± 6.47			4.33 ± 2.83			4.44 ± 2.24			4.78 ± 1.30			4.56 ± 1.67			6.00 ± 2.12		
Unemployment	24.11 ± 5.90			5.29 ± 2.69			3.25 ± 1.58			4.19 ± 1.81			3.71 ± 1.86			7.68 ± 1.82		
Others	25.90 ± 5.42			6.05 ± 2.21			3,65 ± 1.69			4.35 ± 1.78			4.25 ± 1.41			7.60 ± 1.43		
**Family Income**^**a**^																		
< RMW	24.33 ± 5.76	1.23	0.218	5.30 ± 2.64	0.33	.740	3.35 ± 1.62	1.07	.278	4.16 ± 1.77	−0.85	.398	3.79 ± 1.82	1.01	0.315	7.73 ± 1.79	2.31	.025*
> RMW	23.83 ± 5.93			5.24 ± 2.70			3.23 ± 1.56			4.27 ± 1.83			3.66 ± 1.83			7.43 ± 1.95		

Note: ^a^Independent *t*-test was performed, ^b^One-way ANOVA was performed, RMW = Monthly Regional Minimum Wage, ***P < 0.001; **P < 0.01; *P < 0.05.

The differences in mean scores of EBPs according to the characteristic group can also be seen in Table 2. There were significant differences in the mean scores of total EBPs among genders (p < 0.001), areas of residence (p = 0.004), and father's occupations (p < 0.001). On the emotional problems subscale, there were significant differences among genders (p < 0.001), father's occupational (p = 0.001), and mother's occupations (p = 0.035). On the conduct problems subscale, significant differences were found among genders (p < 0.001), father's occupations (p = 0.043), and mother's occupations (p = 0.033) too. On the hyperactivity-inattention subscale, a significant difference was found only between genders (p = 0.028). On the peer problems subscale, significant differences were found among age groups (p = 0.011), areas of residence (p = 0.001), and father's occupations (p = 0.020). Finally, on the prosocial behavior subscale, significant differences were found among genders (p = 0.033), father's occupations (p = 0.021), and household income groups (p = 0.025).

### 3.3 Correlations of EBPs with parental attachment and self-esteem

The mean scores of the father's attachments (M = 82.86, SD = 11.03), mother's attachment (M = 85.11, SD = 9.59), and self-esteem (M = 28.24, SD = 4.18) are shown in Table 3. Table 3 also shows the results of the correlation test. EBPs were significantly negatively related to father's attachment (r = −0.191, p < 0.001), mother's attachment (r = −0.241, p < 0.001), and self-esteem (r = −0.437, p < 0.001). At the same time, parental attachment was positively related to self-esteem (father: r = 0.407, p < 0.001 and mother: r = 0.406, p < 0.001).

**Table 3:** Descriptive statistics and correlations among study variables.

Variables	Mean	SD	1	2	3	4	5	6	7	8	9					
1. EBPs Total	24.01	5.82	1													
2. EBPs Emotional Problems	5.20	2.69	.838***	1												
3. EBPs Conduct Problems	3.29	1.60	.508***	.255***	1											
4. EBPs Hyperactivity	4.21	1.78	.647***	.461***	.327***	1										
5. EBPs Peer Problems	3.68	1.79	.620***	.402***	.352***	.284***	1									
6. EBPs Prosocial	7.62	1.86	.205***	.089**	−.182***	−.145**	−.123**	1								
7. Father's Attachment	82.97	11.03	−.191***	−.244***	−.147**	−.216***	−.206***	.289***	1							
8. Father's Trust	38.18	8.22	−.270***	−.296***	−.230***	−.256***	−.267***	.267***	.910***	1						
9. Father's Communication	29.70	4.28	−.263***	−.320***	−.153***	−.265***	−.238***	.267***	.930***	.819***	1					
10. Father's Alienation	15.09	7.12	.415***	.417***	.258***	.328***	.313***	−.148***	−.551***	−.704***	−.698***	1				
11. Mother's Attachment	81.63	7.39	−.241***	−.258***	−.140***	−.282***	−.219***	.255***	.583***	.530*	.540***	−.324***	1			
12. Mother's Trust	38.44	4.54	−.337***	−.346***	−.230***	−.329***	−.291***	.259***	.548***	.582***	.517***	−.441***	.864***	1		
13. Mother's Communication	29.28	5.42	−.345***	−.364***	0.169***	−.349***	−.297***	.230***	.584***	.558***	.602***	−.469***	.868***	.762***	1	
14. Mother's Alienation	13.91	5.94	.440***	.450***	.226***	.349***	.341***	−.131***	−.387***	−.484***	−.461***	.610***	−.412****	−.638***	−.694***	1
15. Self-Esteem	28.25	4.12	−.437***	−.520***	−.134***	−.331***	−.320***	.171***	.407***	.377***	.472***	−.421***	.406***	.429***	.476***	−.449***

Note: Spearman's correlation test was performed, ***P < 0.001; **P < 0.01; *P < 0.05. Bonferroni correction was performed.

## 4 Factors influencing EBPs among adolescents

Table 4 illustrates the factors affecting EBPs and all subscales. These results were obtained by linear regression analysis for each characteristic and study variable. All predictors of overall EBPs among adolescents were able to explain 31 % of the variance in EBPs according to regression analysis. Overall EBPs among adolescents were associated with father's education (β = −0.08, p < 0.05), father's communication (β = 0.18, p < 0.01), father's alienation (β = 0.27, p < 0.001), mother's alienation (β = 0.18, p < 0.001), and self-esteem (β = 0.27, p < 0.001). Emotional problems were associated with gender (β = 0.26, p < 0.001), father's alienation (β = 0.19, p < 0.01), mother's alienation (β = 0.18, p < 0.001), and self-esteem (β = −0.29, p < 0.001). The conduct problems were associated with gender (β = −0.20, p < 0.001), father's alienation (β = 0.17, p < 0.001), mother's trust (β = −0.15, p < 0.05), mother's communication (β = 0.15, p < 0.05), and mother's alienation (β = 0.17, p < 0.01). Hyperactivity was associated with father's alienation (β = 0.20, p < 0.001), mother's trust (β = −0.12, p < 0.05), and self-esteem (β = −0.21, p < 0.001). Peer problems were associated with gender (β = −0.10, p < 0.01), class level (β = −0.11, p < 0.05), residence status (β = 0.08, p < 0.05), family income (β = −0.08, p < 0.05), father's alienation (β = 0.14, p < 0.01), and self-esteem (β = −0.21, p < 0.001). The prosocial was associated with gender (β = 0.14, p < 0.001), residential status (β = 0.08, p < 0.05), family income (β = −0.10, p < 0.05), father's alienation (β = 0.17, p < 0.05), and mother's trust (β = 0.17, p < 0.01).

**Table 4:** Predictors of EBPs.

Variables	EBPs Total		EBPs Emotional Problems		EBPs Conduct Problems		EBPs Hyperactivity		EBPs Peer Problems		EBPs Prosocial	
	**B**	**β**	**B**	**β**	**B**	**β**	**B**	**β**	**B**	**β**	**B**	**β**
Gender	.752	.064	1.436	.265***	−.669	−.209***	−.173	−.048	−.381	−.106**	.539	.144***
Age	.284	.048	−.017	−.006	.115	.071	−.076	−.042	.119	.065	.142	.075
Class level	−.274	−.038	.081	.024	−.111	−.056	.130	.058	−.258	−.116*	−.116	−.050
Residence Status	1.607	.059	.166	.013	−.070	−.009	.052	.006	.718	.086*	.741	.086*
Father's Education	−.572	−.082*	−.194	−.060	−.104	−.054	−.102	−.048	−.111	−.052	−.060	−.027
Mother's Education	.247	.037	.100	.032	−.043	−.024	.052	.025	.000	.000	.139	.066
Father's Occupation	−.120	−.052	−.004	−.004	−.022	−.034	−.016	−.023	−.505	−.069	−.028	−.037
Mother's Occupation	.008	.003	−.006	−.004	−.025	−.032	.038	.043	−.005	−.005	.005	.006
Family Income	−.896	−.075	−.056	−.010	−.164	−.050	.031	.008	−.303	−.082*	−.404	−.105*
Father's Trust	.004	.005	.001	.003	−.023	−.101	.009	.036	−.006	−.026	.023	.088
Father's Communication	.145	.184**	.042	.115	.025	.118	.018	.076	.014	.059	.045	.177*
Father's Alienation	.357	.278***	.114	.193***	.061	.174**	.079	.202***	.058	.147**	.044	.107
Mother's Trust	−.066	−.061	−.003	−.005	−.045	−.152*	−.042	−.128*	−.036	−.109	.059	.172**
Mother's Communication	−.009	−.009	−.016	−.036	.041	.153*	−.026	−.088	.000	.000	−.007	−.023
Mother's Alienation	.259	.183***	.118	.181***	.070	.179**	.029	.068	.043	.100	−.001	−.002
Self-Esteem	−.370	.272***	−.189	−.299***	−.022	−.058	−.089	−.214***	−.090	−.215***	.019	.044
Constant	19.998		4.680		3.161		7.829		5.494		−1.165	
R^2^	0.310		0.394		0.150		0.222		0.199		0.118	
Cohen's f	0.670		0.806		0.420		0.534		0.835		0.366	

Note: ***P < 0.001; **P < 0.01; *P < 0.05. Bonferroni correction was performed.

## 5 Discussion

In the present research, it was discovered that the bonds with both mothers and fathers had an adverse connection with EBPs while exhibiting a positive correlation with self-esteem. A previous investigation reinforces our findings by demonstrating a harmful link between parental attachment and mental health concerns [10]. Another study revealed that parental hostility was linked to heightened hyperactivity difficulties and trauma symptoms, as well as increased behavioral problems, but only in adolescents who already had significant baseline issues. Conversely, parental warmth was linked to reduced trauma symptoms [39,40]. In line with our current study, research conducted previous study [41,42], similarly demonstrated a connection between limited parental connection and an escalation in mental health problems, particularly in terms of overall difficulties.

Moreover, in line with the findings of the current study, another longitudinal investigation has demonstrated that lower perceived attachment to both mothers and fathers during childhood predicts a greater severity of mental health problems [[43], [44], [45]]. The perception of inadequate parental attachment during childhood may hinder the development of skills to nurture and resolve conflicts within intimate relationships in a healthy and mature manner. Furthermore, a lower perceived childhood attachment to parents could lead to a higher likelihood of misinterpreting the perceptions, emotions, and intentions of significant others, potentially resulting in increased depressive symptoms in adulthood [46,47]. These findings have significant implications for the design of interventions and treatment plans for individuals struggling with behavioral difficulties [48].

Although the present study uncovered a connection between parental attachment and self-esteem, a hierarchical regression analysis demonstrated that father's and mother's attachment factors were individually linked to behavioral issues. This suggests that while these processes are related, they remain distinct factors. Furthermore, the results of this study indicated that father's attachment did not exhibit a significant association with the SDQ (Strengths and Difficulties Questionnaire) among adolescents (β = −0.02, t = −0.81, p = 0.41), despite a correlation between the two variables (r = −0.13, p < 0.001). This finding aligns with previous research that has shown a negative correlation between father's attachment and mental health problems in adolescents [49]. It's important to note that determining the direction of effects in this study is challenging due to its correlational approach.

According to the results of the linear regression analysis, self-esteem exhibited a stronger and more significant association with mental health status and was better at explaining the variance in mental health scores. Self-esteem plays a pivotal role in the development of various mental health conditions. In the context of adolescents, previous research has indicated that low self-esteem is linked to depression during adolescence and young adulthood, whereas high self-esteem tends to be connected with greater well-being and overall life satisfaction. Low self-esteem among adolescents has consistently been associated with mental health issues. Furthermore, multiple prior studies have demonstrated the relationship between teenagers' self-esteem and their mental health [[50], [51], [52]]. In terms of mental health, self-esteem is a crucial factor and has been found to be associated with mental well-being [[53], [54], [55], [56]].

This study confirms the widely held belief that adolescents who have stronger bonds with their parents and higher self-esteem tend to experience fewer overall challenges and exhibit more positive prosocial behavior. However, it's important to highlight that not all of these variables exhibited direct connections with mental health outcomes when considered simultaneously in the same regression model. The study underscores that not all aspects of parental attachment directly influence the mental health of adolescents, with maternal attachment emerging as the primary influential factor.

The practical implications of this research are significant, especially for the development of interventions and treatment strategies aimed at enhancing the mental well-being of adolescents. Recognizing the critical roles played by maternal attachment and self-esteem can guide targeted interventions designed to bolster these factors, potentially leading to more favorable outcomes for adolescents in terms of their behavior and emotional health.

### 5.1 Limitation

From a methodological point of view, attention should be called to the specifics of the sample. The data collection was only in one school, located in the south of Padang City. It can be assumed that compared to other regions in Padang, this area, more adolescents grew up in well-educated, better-off families. Sampling hence is likely to have contributed to our specific results. Further, the cross-sectional design limited the ability to make causal inferences.

## 6 Conclusion

This study aimed to explore the association of parental attachment and self-esteem with mental health in adolescents. The study showed that adolescents with more stable relationships with their parents and strong self-esteem had a number of positive mental health outcomes. This study's findings support the need to develop numerous attachments, secure attachments, and strong self-esteem during adolescence. Although there was a correlation between variables, it is unclear whether lower adolescent attachment to their parents was a precursor to whether individuals who exhibited poorer mental health had an incredible difficulty forming attachments. Although long-term studies are needed to support this claim, some researchers have hypothesized that the links between attachment security and mental health are reciprocal. The cross-sectional design limited the ability to draw causal conclusions.

## Data availability statement

The data is not accessible to the public as it contains information that might compromise the confidentiality of the research participants. Nevertheless, the data can be made available upon a reasonable request.

## Funding

This study was supported by Research and Community Service Centre (Lembaga Penelitian dan Pengabdian Masyarakat) Fund of the 10.13039/501100014563Universitas Andalas, Padang, Indonesia (Grant Number T/155/UN.16.17/PT.01.03/KO-RPT/2022).

## CRediT authorship contribution statement

**Rika Sarfika:** Conceptualization, Data curation, Formal analysis, Funding acquisition, Investigation, Methodology, Project administration, Resources, Software, Supervision, Validation, Visualization, Writing – original draft, Writing – review & editing. **I Made Moh Yanuar Saifudin:** Data curation, Formal analysis, Investigation, Software, Validation, Visualization, Writing – original draft, Writing – review & editing. **Ira Mulya Sari:** Data curation, Project administration, Writing – original draft. **Dewi Murni:** Resources, Writing – original draft. **Hema Malini:** Writing – review & editing, Formal analysis. **Khatijah Lim Abdullah:** Data curation, Writing – review & editing.

## Declaration of competing interest

The authors declare that they have no known competing financial interests or personal relationships that could have appeared to influence the work reported in this paper.
